# Political instability and hiv/aids response in the south west and north west regions of Cameroon: a qualitative study

**DOI:** 10.1186/s12889-023-16994-w

**Published:** 2023-11-03

**Authors:** Julius Enongene Mekolle, Katayi Edouard Tshimwanga, Niba Juste Ongeh, Agbor Nyenty Agbornkwai, Omeichu Agwenam Amadeus, Ismaela Esa, Keshia Ebude Mekolle, Ndung Ako Forbinake, Claude Ngwayu Nkfusai, Pascal Nji Atanga

**Affiliations:** 1 Family Health International, Bafoussam, Cameroon; 2 Africa Center for HIV/AIDS Management, Stellenbosch University, Stellenbosch, South Africa; 3 Cameroon Baptist Convention Health Services, Bamenda, Cameroon; 4 Clinical Research Education Networking and Consultancy (CRENC), Yaoundé, Cameroon; 5 Universite Catholic d`Afrique Centrale, Yaoundé, Cameroon; 6 World Health Organization, Buea, Cameroon; 7 School of Nursing and Public Health, University of KwaZulu- Natal, Durban, South Africa

**Keywords:** Cameroon, HIV/AIDS, Political instability, Global health, Qualitative health research

## Abstract

**Background:**

Politically motivated violence and insecurity continue to be a major threat to progress in HIV epidemic control and a significant contributor to health inequality. Despite a decreasing HIV/AIDS disease burden, the Republic of Cameroon in West Africa is experiencing ongoing political instability in her North and South West Regions. Our study used qualitative methods to better understand key frontline health care providers’ (fHCP) perceptions of the effects of political instability on HIV/AIDS response gains in Cameroon since 2018, as well as lessons learned for overcoming them.

**Methods:**

Between July and August 2022, semi-structured, in-depth key informant interviews involving 10 purposively selected participants were conducted in the two regions. Interviews were recorded and transcribed verbatim, coded thematically, and analyzed manually.

**Results:**

Six overarching themes emerged from the transcribed key informant interviews. They were as follows: Challenge with access to and availability of health care services (HIV care, commodity supply chain) in the smaller communities; Impact on continuity of treatment; Increased risk of new HIV infections; The socioeconomic impact of the crisis on the quality of life; The impact of the crisis on fHCPs’ physical and mental health and the health system’; and Coping mechanisms.

**Conclusions:**

Frontline healthcare workers have acknowledged the significant impact the current political instability has had in hindering the development and advancement of a successful local response to HIV/AIDS in the two impacted regions of Cameroon. Coordinated efforts must be made to strengthen the health sector in areas such as HIV healthcare decentralization, supply chain, and protecting frontline healthcare workers from political quagmires in order to lessen the impact of the nation’s socio-political crisis on the HIV/AIDS response and, more generally, on the entire health sector.

## Introduction

### Background

The current political crisis in Cameroon’s North West (NW) and South West (SW) regions is a particularly concerning recurrence of a long-standing issue. Never has there been such a high level of anxiety about the Anglophone regions` issue [1]. The situation began as peaceful protests in 2016, but quickly evolved into a full-fledged armed conflict. This has prompted responses in the areas of security, humanitarian aid, and politics. In response to the security challenges, the government intervention was prompt, and accounts suggest that this early militarization intensified the conflict by radicalizing separatist groups and strengthening their determination [2]. The two affected regions are home to some 68,678 persons living with HIV [3]. With the emergence of the political unrest in the setting, there now seem to be a new context for the person-centered adaptation of HIV care services [4–7].

During conflicts, disruptions in health service delivery increase the risk of preventable mortality and morbidity among the affected population [8, 9, 10], and armed conflict victims experience a variety of traumas, which can be physical, psychological, or both [8]. The crisis has had far-reaching consequences; during the armed conflict, the populace of the Southwest region utilized less health services primarily because of difficulty accessing health facilities [11]. This might have a substantial impact on the population’s health, other global health targets such as enhanced vaccination uptake [10, 11] and coverage in all health districts as well as the UNAIDS 95-95-95 agenda for HIV/AIDS epidemic control [11], which states that 95% of people with HIV know their HIV status, 95% of those who know they have HIV are on lifesaving antiretroviral treatment, and 95% of those who are on treatment are virally suppressed [12]. With the paucity of data exploring the relationship between political conflicts and HIV response in sub-Saharan Africa, especially in Cameroon, the concept seems to attract limited attention for policy change. Moreover, there are no existing Cameroonian national guidelines guiding approach towards navigating HIV services in conflict settings.

Cameroon, located in Sub-Saharan Africa has a population of about 27 million [13], and an estimated 534,245 people living with HIV among whom 56.68% (302,822) were receiving ART by the end of June 2019 [14]. The epidemiological burden of HIV in Cameroon is decreasing (from 5.5% to 2004 to 4.3% in 2011 to 2.7% in 2018) [15]. This progress is largely due to the concerted efforts of the government of Cameroon and its technical and financial partners in implementing evidence-based innovative strategies such as the rollout of PMTCT option B + in 2013, the “Test and Treat” strategy since 2016, increasing viral load uptake and coverage since 2007 [16], and the elimination of user fees for essential HIV-related services. Despite these gains, the ongoing political instability can quickly overturn many years of committed hard work [17]. Against this backdrop, we aim to shed light on frontline health workers’ perceptions of the effects of political instability on the HIV/AIDS response in the NW and SW regions of Cameroon and lessons learned for overcoming these obstacles. A deeper understanding of the inter-tangled relationship between political instability and the HIV/AIDS response may offer insights into the contextual impact of such a complicated relationship and may potentially suggest new strategies for strengthening resilience, as well as add to current knowledge in the field of HIV and public health.

The general objective of this study was to investigate and document the impact of the socio-political crisis on the HIV response in Cameroon`s two affected regions. The specific Objectives included:

- To understand the perceptions of frontline healthcare workers in the South West and North West Region of Cameroon on the effects of the ongoing socio-political unrest since 2018 in relation to HIV services.
- To investigate their experiences on approaches and recommendations to cope with the HIV/AIDS epidemic amidst the country’s current political instability.

## Methods

### Study design

We adopted a qualitative study design using the phenomenological approach [18] to understand frontline healthcare workers’ perceptions of the impact of chronic political instability on the HIV/AIDS response in the South West Region and North West Region between 2018 and 2022. Participants were interviewed between July and August 2022.

### Participant selection

The selection of key informants was guided by the desire to recruit a cream of healthcare providers with first-hand witness, based on their responsibilities, experiences, and expertise in the HIV/AIDS response in Cameroon between 2018 and 2022. Using the purposive and snowball sampling methods [19], we recruited a total of ten key informants. We included:1) health care workers with at least 2 years of residing and providing services permanently in communities affected by the political unrest prior to and during the crisis; 2) availability and willingness to give informed consent to participate in the study. The roles that each informant played in the HIV response at the managerial and health facility levels included carrying out the UNAIDS 95-95-95 agenda [12]; improving service quality and ensuring that deliverables are completed on schedule.

### Data collection

We used an open-ended semi-structured interview guide to better understand the perception of key frontline healthcare providers of the impact of political unrest on the HIV/AIDS response from 2018 to 2022 in the NW and SW regions. The interview guide was developed based on firsthand accounts of Cameroon’s HIV/AIDS response. The interviewees had access to the printed English version of the guide both prior to and during the interview. Because the interviews were semi-structured, the interviewer was able to customize the order of the questions and make use of probing questions to the key informant’s particular function, background, or area of expertise. The interview guide’s goal was to serve as a starting point for prompts to be used in order to get complete, flowing, and natural responses from informants rather than systematically asking each question in the guide.

Six of the ten interviews were administered face to face, while four were conducted over an encrypted phone (Xiaomi Redmi Note 8). Audio data obtained were transferred and stored on an encrypted laptop, accessible only to the PI. All interviews were finally conducted in English. Interviews involved 3 informants from the NW and 7 from the SW regions. Among the interviewees, 3 were from faith-based facilities and 7 from government-owned health institutions. Interviews lasted between 15 and 52 min (average of 32 min). Verbatim transcriptions of every key informant interview were made. The interview notes were used as a supplement to the transcriptions that served as the main source of data. A transcription file with about 32,000 words kept in the original language was produced. Nevertheless, the overall quality and depth of the interviews conducted were prioritized over their quantity.

In accordance with the “data saturation” paradigm proposed by Saunders et al., we concluded that saturation had been reached during data collecting when no new themes emerged [20]. Respondents were able to speak uninterrupted thanks to the open-ended semi-structured format. The verbatim transcription has the added benefit of producing a complete transcription file which allowed for thematic analysis.

### Data analysis

This research was both inductive and iterative. Thematic analysis [4, 21] was performed manually on verbatim transcripts in Microsoft Word. We carried out coding by assigning labels to lines of text while classifying and contrasting comparable or related bits of data. Themes and sub-themes were created by organizing and categorizing the codes. Before beginning the coding process, the transcripts were collected into a single file and reviewed many times. A consensus-based reading frame of the transcript data was created, themes and sub-themes were coded, and competing perspectives were iteratively settled. The evolved topics were described in a narrative form. Variations in opinions were detected in the areas where they stood out the greatest. The themes and sub-themes were contextually brought to life with the use of pertinent supporting quotations. The findings were organized into recurrent themes with multiple sub-themes for each theme that resulted from the data analysis. Returning to key informants and asking for clarifications when the interviewer had questions helped to ensure quality control.

## Results

As summarized in Table 1 below, six overarching themes emerged from the transcribed key.

informant interviews, as follows: Challenge with access to and availability of health care services (HIV care, commodity supply chain) in the smaller communities; Impact on continuity of treatment; Increase risk of new HIV infections Socio-economic impact of the crisis affecting quality of life; Impact on physical wellbeing and mental health of HCPs and health system; Coping mechanisms.

**Table 1:** Summary of main themes, subject themes, and supporting evidence

Main theme	Subject theme	Supporting evidence
**Challenge with access to and availability of health care services (HIV care, commodity supply chain) in the smaller communities**	Erratic lockdowns	*“…during the lockdown period, it was an interesting scene to see a pregnant lady brought into the hospital by an armored military vehicle. It happens she has been very ill for long and they’ve trekked for miles without a means of transport”* *‘…we observed some really severe anemias in children and worrying advanced diseases because patients could not make it to the health facility timely enough. Even referral of these patients to tertiary hospitals was a problem’*
	Displacement of community-based HCP for fear of their lives	*‘…the HCPs have to run for their lives so access to health care services in the smaller communities not only HIV became a big challenge…’*
	Insecurity as a driver to constraints in access	*‘…almost 75% of our clients are in the community…as long as the crisis continues to intensify, if we don’t have means to go to the community those people are likely to die.*
	Shut down or destruction of health facilities offering decentralized minimum package of HIV care services	*‘…and with the intensification of the crisis some of these HF has to shut down’* *‘…my hospital was the only referral health facility in the division. 9/12 blocks were completely set ablaze. You can imagine the position this leaves patients, both acute and chronic especially PLWHA.*
**Impact on continuity of treatment**	Insecurity as a driver of altered priority complicates the already existing barrier to health care	*‘…the people are locked in…they feel like they are fine so why should they take that risk to come for medication…a patient will tell you…a gunshot will kill me faster than me not on drugs’*
	Drop in client active file number as an impact of the crisis	*‘…but immediately the crisis started you see the active file dropped drastically from the 3950 to about 3100…’*
	Crisis-induced economic constraint as an intercalating syndemics driver to missing appointments	*‘…due to economic hardship and clients are unable to come to come to the treatment center’* *‘…You have people coming here leaving areas where you pay as much as 20.000 FRS for only one-way transport to come to the treatment center. So all of those people at that time were unable to come…’*
**Increased risk of new HIV infections**	Missed opportunities at ANC and reproductive health	*‘…children born in very difficult conditions …not delivered by trained HCP increase the rate of MTCT in these communities…’*
	Increasing new cases from remote communities	*‘…most of the new cases are coming from very remote communities and these are people who have not had contact with HCP for more than 3–4 years…and also some of the communities have not benefited from community outreach where they could receive education on testing, prevention* *‘…People now live in camps favoring promiscuity’*
	Migration as a predisposing factor to HIV spread	*‘…and because of the displacement and other factors they may have been involved in a lot of activities that put them in a compromising situation…’*
	Power interplay as a driver of gender-based vulnerability in the crisis	*‘…some of them were raped …they`ll tell you - I did not consent to it but could not refuse because he has a gun, he takes care of my family so even though I am not comfortable with what I am doing I just have to for safety of my family…’*
**Socio-economic impact of the crisis affecting quality of life**	Economic constraint as a driver to unhealthy livelihood	*‘…People including PLWHIV have become displaced and are now perching in bushed and other safe heavens. This has made cost of living very expensive leading to poor nutrition for PLWHIV’*
	Women taking up breadwinner roles	*‘…some have lost breadwinners of their family…many women have lost partners who were breadwinners, some widows have lost children who could be the source of livelihood to them’*
**Impact on physical well-being and mental health of HCPs and health system**	Healthcare providers as targets in the crisis	*‘…so many HCP have been victims…kidnapped, beaten, tortured, some killed just for them being available and doing their jobs…this alone has affected every HCP psychologically…’*
	Arson on hospital premises is a strong measure of insecurity even in the healthcare institution.	*‘…Arson attack on my hospital was a very strong statement. Many HCP who are supposed to be caring for patients lost their jobs…’*
	HCP absenteeism and abandonment of duty	*‘…we had health workers who abandon their posts…’*
**Coping mechanisms**	Reinforcement of client counselling and education	*‘…so the first thing we have learned is to empower the client with the information, and make them to take responsibility for their health…’*
	Clients engagement through the creation of CAGs	*‘… we have had the opportunity to encourage clients to be one another’s keeper…through creation of CAGs…’*
	Facility-led community- based approach	*‘…my team planned from the facility trips to the community…’*

## Perceptions of frontline health care workers in the South West and North West Region of Cameroon on the effects of the crisis or its impact on the quality of HIV healthcare delivery since 2018

### Challenge with access to and availability of health care services (HIV care, commodity supply chain) in the smaller communities

Constraint in the availability of and access to health care service in all its ramifications was a very popular repercussion of the crisis reportedly experienced by the population which immediately affected HIV services. Respondents portrayed the effect to be directly related to erratic lockdowns, displacement of community-based health care providers (HCP) for fear of their lives, insecurity as a driver to constraints in access, shut down or destruction of health facilities offering decentralized minimum package of HIV care services and disruption in ARV supply and dispensation practices.

#### Erratic lockdowns

Informants said frequent spontaneous or planned lockdowns created multiple problems with the availability of and access to HIV-related health services. Lockdowns led to restrictions in smooth movement preventing patients from the uptake of services.

*‘…during the lockdown period, it was an interesting scene to see a pregnant lady brought into the hospital by an armored military vehicle…they’ve trekked for miles without a means of transport.’*

As a result of problems with access to ARVs some patients tend to adopt other measures to adapt while looking forward to the closure of the lockdown.

*‘…we are told stories of how PLWHA who know themselves or who share the same status in families share ARVs among themselves until the last tablet simply because of road blockage in the crisis.’*

Commonly reported by the respondents was the fact that patients known to have been ill at home for long periods secondary to constraints in access, only visit the health facility in severe health states:

*‘…we observed some really severe anemias in children and worrying advanced diseases because patients could not make it to the health facility timely enough. Even referral of these patients to tertiary hospitals was a problem.’*

#### Displacement of community-based health care providers for fear of their lives as a worsening indicator widening the health care service availability gap

Some respondents saw the fact that health care providers fleeing for their lives complicated access to and availability of optimal care for the community dwellers, worse still as they cannot travel to seek this care elsewhere due to either insecurity of travel or lockdowns.

*‘…the health care providers have to run for their lives so access to healthcare services in the smaller communities not only HIV became a big challenge…and they can’t travel to further distances (for health care)….’*

*‘…health care workers and community health workers who provided HIV healthcare service delivery have fled; unfortunately they were the ones who knew the clients in detail including residential localization.’….*

#### Insecurity as a driver to constraints in access

Insecurity clearly restricts any form of movement. In some hard-to-reach communities where health care providers had to make regular trips for ARV dispensation, insecurity prevented circulation of both HCP and PLWHA, hence access to HIV care was greatly affected.

*‘…almost 75% of our clients are in the hard-to-reach communities such as creeks… as long as the crisis continues to intensify, if we don’t have means to go to the community those people are likely to die. If there is no means of access due to worsening kidnaps, gunshot exchanges, militarization of the community, those people find it difficult to come to the facility and PLWHA will likely develop worse health states.’*

#### Health facilities offering decentralized minimum package of HIV care services were shut down or destroyed

Prior to the crisis HIV care services by ministerial order were decentralized to some peripheral health facilities [22]. Some respondents reported shutdown of health facilities thereby overturning this vital effort toward bringing care closer to the local population. Arson of hospital building was also reported to be a major deterrent to health care access.

*‘…and with the intensification of the crisis some of these health facilities have to shut down.’*

*‘…my hospital was the only referral health facility in the division. 9/12 blocks were completely set ablaze. You can imagine the position this leaves patients, both acute and chronic especially PLWHA. I recall that among others, all viral load samples collected and preserved were completely destroyed in the process…Luckily the treatment unit was untouched, so we bravely tiptoed across the ruins to serve the courageous clients who came to pick up their drugs. However, the incident had created a terrifying atmosphere for most clients, plus of course other key complementary services such as PMTCT and advanced disease management requiring hospitalization were now unavailable.’*

#### Disruption in ARV supply and dispensation practices

Informants described supply chain of HIV- related commodities such as ARV as one of the areas affected by the crisis. Supply chain constraints heavily affected by insecurity meant drugs, condoms, viral load sample collection materials etc. could not reach health facilities timely enough. The resultant effect of the non-availability of ARVs includes stock-outs. Dispensation practice such as multi-month dispensation was ignored, making clients having to visit the facility multiple times against a backdrop of insecurity.

*‘…even within this period, the supply of ARV have not been like it used to be. We have at times so many stock-out situations, even for the drug to get to the center it has been a very big problem….’*

*‘…and not only bringing…also transporting the drugs from the regional level to the treatment unit was not and even up to now is not easy…most of the supply drivers did not just want to ply the roads because of the nature of the crisis… but this was not the case before the crisis because before the crisis it was easy, the ambulance could go (pick up the drugs) but during the crisis we know even the lone ambulance we had was roasted somewhere along the road…making it difficult for us to meet up…’*

*‘…and then the ARVs were not in their quantities so we had to dispense only what we have…before the crisis most clients were used to multi-month dispensation, with the crisis and the stock out situation, we were unable to dish them multi-month dispensation, we only gave 1 month and some clients will stay back 1 or 2 months before coming due to insecurity and so you see a real effect on activities in treatment center….’*

### Impact on the continuity of treatment

Several respondents conceptualized that keeping PLWHA on treatment in the crisis era was an uphill task with clear direct or indirect interconnectedness between the crisis and continuity of treatment. They reported that factors involved include; insecurity as a driver of altered priority complicating the already existing barrier to health care, erratic lockdowns, crisis-induced economic constraint as an intercalating syndemics driver to missing appointments, spontaneous emergency temporal and permanent migration of clients due to harsh living environment, abandonment of treatment, displacement, loss of contact and challenge for tracking by the health facility. One interviewee summarized it as thus:

*‘…but immediately the crisis started you see the active file dropped drastically from the 3950 to about 3100….’*

#### Insecurity as a driver of altered priority complicating the already existing barrier to healthcare

Insecurity was commonly echoed to serve as a major progenitor of multiple repercussions of the crisis on HIV response, including adversely affecting the continuity of treatment. Because clients were afraid of circulating, they tend to miss treatment, become defaulters or interrupt their treatment voluntarily or involuntarily.

*‘…before the crisis the number of treatment centers that existed in the region were even quite limited…and now with the socio-political crisis it became more challenging because movement has been a very difficult thing due to safety reasons…coupled with the risk that was at different junctions getting to the hospital to pick their ARVS.’*

Reportedly, client would clearly re-order their priority for survival putting security above their apparent good health as testified by one informant.

*‘…the people are locked in…they feel like they are fine so why should they take that risk to come for medication…a patient will tell you…a gunshot will kill me faster than me not on drugs.’*

#### Crisis-induced economic constraint as an intercalating syndemics driver to missing appointments

The socio-political crisis was reported to affect the denizens economically. Several informants shared that this has a ripple effect on the health-seeking behavior of PLWHA especially regarding the pick-up of their medications.

*‘…due to economic hardship clients are unable to come to the treatment center.’*

*‘…you know the treatment center in DHK is not only town-based. Most of the clients are coming from the suburbs. You have people coming here leaving areas where you pay as much as 20.000 FRS for only one-way transport to come to the treatment center. So all of those people at that time were unable to come…’*

#### Spontaneous temporal and permanent migration of clients due to harsh living environment

The crisis made living conditions precarious for people. In response to erratic insecurity incidences and the need for better living conditions they continuously are on the move. One respondent attested to the fact that the spontaneous migration has affected the continuity of treatment of their cohort, as some clients even abandoned their treatment. Another reported loss of ARVs in the process of a client seeking immediate safety. Confidentiality and perceived stigma were notably a cause of concern as PLWHA migrate to a new location.

*‘…clients actually move, they change location, some of them left their homes to bushes…transferred to other towns…other villages and so it was very difficult for them to come to the clinic, I think that is the main reason…and some even abandon the treatment.’*

*‘Persons fleeing into the bushes may lose their ARVs in the process, many have relocated and for confidentiality purpose do not visit any nearby facility for drug refill of salvage.’*

Informants also mentioned how displacement of PLWHA to unknown destinations led to loss of contact which presented a huge challenge for tracking by health facility.

*‘Clients became displaced without any prior notification and proper planning with their service providers. Thus they become defaulters/LTFU.’*

*‘…That alone has caused a lot of clients who were on treatment to either be irregular or interrupt treatment and we are unable to trace them because they have lost livelihood, and contact information and have been displaced internally’….*

#### Erratic lockdowns

Imposed restrictions in circulation meant that clients were unable to travel to health facilities for medication pickup. Community health workers as well were unable to make trips for community dispensation to hard-to-reach areas. This clearly negatively affected the continuity of treatment for PLWHA as intimated by a respondent.

*‘…Continuity of treatment is greatly affected due to many days of ghost towns with movement restrictions.’*

### Increased risk of new HIV Infections

Nearly all respondents mentioned an increase in the risk of new HIV infections as a result of the political unrest, with power struggles serving as a particular driver of gender-based vulnerability in the crisis that exposed young girls and teenage women. Sub-themes were repeated around missed opportunities at ANC and reproductive health, hard-to-reach nature of remote communities and migration as a predisposing factor to HIV spread.

#### Missed opportunities at ante-natal care and reproductive health

Ante-natal care is a key entry point in the prevention of mother-to-child- transmission (MTCT) of HIV. Many informants mentioned that this important service including reproductive health was lacking with the emergence of the crisis particularly in the remote communities.

*‘…so this has led to (we can say that) an increase in MTCT of HIV because we have had situations where we see children who were born of HIV positive pregnant women and did not have the privilege to attend ANC and we only saw them when they were sick and they could manage to bring them to hospital so they did not receive any intervention during pregnancy or early delivery stages’….*

*‘…children born in very difficult conditions …not delivered by trained HCP increase the rate of MTCT in these communities’…*.

…secondly you see with the female, the younger population…between 20 and 35 most of them were out of treatment and are the population that moved most….

#### Hard-to-reach nature of remote communities

The hard-to-reach nature of some communities was seen by some informants as a potential contributor to the risk of new infections. They argued that these communities had not benefited from health care, education and health promotion outreaches during the span of this crisis. Also, they added that the make-shift settlements in the bushes by those who had fled their homes were fertile grounds for propagating promiscuity.

*‘…most of the new cases are coming from very remote communities and these are people who have not had contact with HCP for more than 3–4 years…and also some of the communities have not benefited from community outreach where they could receive education on testing, prevention…so we believe that a lot of new infections have come as a result of this because many people who were supposed to be on treatment have not been on treatment’.*

*‘…people now live in camps favoring promiscuity’*.

*‘…because of this issues…before the crisis you get to the local community and screen only a few people positive, but you now get to the same community you screen more people positive…positives which are even more than national prevalence which in a way justify the effects of the crisis’….*

#### Power interplay as a driver to gender-based vulnerability in the crisis

Majority of respondents spoke of power as a tool encouraging the gender implications of the political instability, remarkably the heightened vulnerability of adolescent girls and young women. The proliferation of transactional sex whereby young women and widows were guaranteed family protection, security and provision for feeding in exchange for sexual relations was raised as an adaptive measure among some denizens.

*‘…another thing that is key…is that the crisis empowered some people and made some vulnerable. Those who carry guns have a lot of power…and the women and girls are vulnerable…there are stories of one man with multiple wives as girlfriends. Sometimes we test women whose partners are key actors in the war and from what they tell us… they are just one among many…if that’s the case, so many people have been exposed and the incidence among this vulnerable group (women and young girls) has increased,… and from the age group of the clients we receive, we discover that some of the young people are getting the infections’….*

*‘…some of them were raped …they’ll tell you - I did not consent to it but could not refuse because he has a gun, he takes care of my family so even though I am not comfortable with what I am doing I just have to for safety of my family…’*.

Gender-based violence (GBV) was also repeatedly reported.

With the political instability, GBV has been on the increase thus increasing the incidence and prevalence of HIV with the female folk being at higher risk.

*‘…an experience is that of a lady who was aware of her status but refused to disclose to her partner for fear of being brutalized by the partner…especially with no one to turn to for support in the context of the crisis….’*

#### Migration as a predisposing factor to HIV spread

Migration was a very common coping mechanism by the community dwellers in response to the discomfort of the political instability. Some respondents shared that this, in a way enhanced risk of new infections.

*‘…and because of the displacement and other factors they may have been involved in a lot of activities that put them at a compromising situation’….*

### Socio-economic impact of the crisis affecting the quality of life

As the crisis worsened, people tended to migrate internally and externally, leaving their stable source of livelihood such as farm produce and businesses to new uncertain settlements. With the spontaneous displacement comes dependence on hosts and socio-economic hardship. This, as fallout of the war, was repeatedly narrated by some respondents to have negatively influenced the HIV response. Discussions on the topic centered on the economic constraint as an intercalating syndemics driver to unhealthy livelihood, and women taking up breadwinner roles. One respondent summarized it thus;

*‘…and so the general state of their economic power has dropped and so it someway affects the quality of their health…they cannot afford transport to the hospital, they cannot pay medical bills when they fall sick, they cannot feed well…and that has also contributed to an increase in the amount of advanced HIV cases we see in the hospital because there are people who have been out of treatment for some time… and so their immune states drop and they have to acquire OI’…*.

#### Economic constraint as an intercalating syndemics driver to unhealthy livelihood

The crisis only came to aggravate the already depleted economic situation of the community dwellers. Nutrition is a key aspect in the health maintenance of PLWHA, yet as a result of economic difficulty many are exposed to nutritional comorbidities. These were clearly articulated by some respondents.

*‘…PLWHA have become IDPs depending on well- wishers for their basic needs, and persons have become orphaned and widows/widowers as a result of the crisis. Also persons (farmers) cannot go to their farms as they would have loved’….*

The crisis has had great consequences on the social aspects on life. People including PLWHIV have become displaced and are now perching in bushed and other safe heavens. This has made cost of living very expensive leading to poor nutrition for PLWHIV.

*‘…some of them will tell you - my business was burnt…there is no way to transport farm produce in exchange for money…some don’t bother to come to the hospital because, how do they transport themselves, some have to spend 40000–50000 FRS and they don’t have that kind of money’….*

#### Women taking up breadwinner roles

In a war where men were mostly targets, women were reported to be disproportionately vulnerable. As the men became victims of casualty in the process, women were taking up roles of leading and sustaining families individually or communally, but not without implications. Some informants reported as described.

*‘…on the other hand, for female, the burden of caring for a family without fathers lost to the war also ways on them psychologically and economically which is affecting their health-seeking behavior including value of HIV’.*

*‘…some have lost bread winners of their family…many women have lost partners who were breadwinners, some widows have lost children who could be source of livelihood to them’*.

#### Impact on physical well-being and mental health of HCPs and health system

When asked about the potential implications of political instability on the health system and health care professionals, a number of respondents characterized those effects as having negative effects on the HIV/AIDS response at every level of the health strata as they rippled through the system. Four subthemes that emerged were: healthcare providers as targets in the crisis; arson of hospital premises as a strong measure of insecurity even in the health care institution; health care provider absenteeism and abandonment of duty; absence of much relevant regular face-to-face mentorship/supervision due to insecurity.

#### Healthcare providers as targets in the crisis

The occurrence of overt and deliberate attacks on healthcare providers during the crisis was not uncommon. Some respondents graphically narrated representations of the unfortunate happenings with mental health and output implications:

*‘…throughout the crisis safety has been the top priority for everyone, so many health care providers have been victims…kidnapped, beaten, tortured, some killed just for them being available and doing their jobs…this alone has affected every health care provider psychologically…because they have been targeted…this has affected the altitude of work…because sometimes in the midst of your patients you don’t feel safe for fear of victimization or being targeted from what you say’*.

*‘…because even the healthcare providers… at one time were targets to some of these groups…for example in my hospital…people have been attacked at the job site’*.

*‘…service providers are not able to execute their duties as required due to insecurity. Frequent gun shots, and kidnappings cause service providers to relocate to safe heavens’*.

#### Arson of hospital premises as a strong measure of insecurity even in the health care institution

At least two informants vividly narrated arson attacks on health facilities, a practice which destroyed health facility space and transmitted fear and frenzy among healthcare workers.

*‘…most of the health workers knew that if things become worse as there are …they’ll finally have shelter in the hospital but when the hospital was burnt down it was the last hope…so people knew that there is nowhere that is safe’*.

*‘…rule of war includes no attack on institutions like hospitals. The arson attack on my hospital involving more than 75% of the building was a very strong statement. Many health care providers who are supposed to be caring for patients lost their jobs. Even those who braved the odds to provide services at the site of the ruins, do so with extreme caution and uncertainty. It is a perpetual mental stress to live and work in this sort of unpredictable environment’*.

#### Healthcare provider absenteeism and Abandonment of duty

As health care workers deal with unstable conditions, informants frequently reported absenteeism and desertion of duty:

*‘…the staff at times…are not at work because …the quarter in which they live is not safe…and so they have to wait until things are calm before they can come (to work), some don’t come to work the whole day and you’ll not have reasons to sanction them because you`ll be getting the sound of the guns…so it affected the health workers…directly or psychologically’…*.

One respondent explained that the outward emigration of specialists and health workers active in the HIV response in search of better living and working conditions in other towns might also be seen as a cascading effect outside of the health system itself:

*‘…prior to crisis, in a community like Kumba, there are hospitals which were equipped with specialist services on a regular basis and some by support visits, but with the crisis those services are no longer available…the competent package of services are no more there so patients have to travel further for these’…*.

*‘…health care providers in the remote communities had to completely flee, and so the population has to struggle…this has also led to provision of services by clandestine or undertrained individuals’….*

#### Absence of much relevant regular face-to-face mentorship/supervision due to insecurity

Some respondents carefully pointed out the impact the crisis also had on attitudes towards mentorship and supervision of HIV activities in the health system:

*‘…but as of 2017 most of these supervisions that were coming down…you know in the treatment center we feel the impact of supervisors when they come and face things directly with you in the field but most could only end at the regional level making it difficult for them to come down to the field…’*

*‘…and so some of the wrongs were difficult to be corrected because all could only be done virtually’.*

## Experiences on approaches and recommendations to cope with the HIV/AIDS epidemic given the country’s current political instability

### Coping mechanisms

When asked about approaches to cope with the HIV/AIDS epidemic given the country’s political instability, respondents often spoke about the importance of reinforcement of client counselling and education, community-based initiatives such as community ART groups, and facility-led community-based approach.

#### Reinforcement of client counselling and education

Some respondents mentioned the need for continuous health education and promotion as a key tool for helping PLWHA cope with the impacts of the crisis. They reiterated the essence of detailed partnership with clients putting them at the focus of their health:

*‘…I’ve learnt a lot of lessons during this period…throughout the period we saw that health care service delivery and especially for chronic care is a team work between the health care provider and the patients… so the first thing we have learnt is to empower the client with the information, and make them to take responsibility for their health. This made us to explore the strength of our patients and also explore opportunities in the communities they’re coming from so that we can mitigate some of the challenges’.*

*‘…in terms of approaches, we have been putting in more in counselling, health education…because at this moment that was the only tool that one could really give because people are losing hope…despite the odds we still increase number of people going out for testing and community dispensation’…*.

*‘…The key strategy is to plan with the clients on how they will receive services in days of lockdown’*.

#### Clients engagement through creation of community ART groups

Informants also reported the adoption of community-based initiatives such as community ART groups as an effective adaptation to service delivery in the face of the crisis.

*‘…we have had the opportunity to encourage clients to be one another’s keeper…through creation of community ART groups, all of these to empower the clients to identify their own health needs, be able to advocate for them to be met’….*

*‘…in the face of the insecurity, we encourage clients to group themselves. One person who has the opportunity to travel picks up treatment for their peers, especially those without ID cards or younger men who are likely targets’.*

#### Facility-led community based approach

The facility-led community-based approach was successfully employed as a model of care in addressing differentiated service delivery for HIV in conflictaffected settings as narrated by one informant.

*‘…despite the insecurity challenges, my team planned from the facility, trips to the community. Adorned in easily identifiable jackets, we went along with ARVs and viral load sample collection materials which we succeeded in testing, dispensing medication and collecting samples of many clients’*.

## Discussion and conclusion

Politically-driven violence and insecurity remains a major threat to gains in the HIV epidemic control, and a potent enhancer of health inequality. This is the experience of the Ukraine crisis [23], further echoed by the executive director of UNAIDS in her 2022 world AIDS day statement [17].

Our study examined the perceptions of frontline health care workers (fHCWs) in the South West and North West Region of Cameroon on the effects of the ongoing political unrest on the quality of HIV health care delivery since 2018. Our findings, which are based on thematic analyses of transcriptions from ten in-depth key informant interviews, show that political instability has had a profoundly negative impact on the residents of these two English-speaking regions. Frontline healthcare workers believe that since the beginning of this instability, there have been significant negative consequences on the country’s HIV/AIDS response that continues to date. Political instability affects all facets of society, including the health sector. Because effects on the health sector have ramifications on people’s lives, they may be more obvious than in other aspects of the HIV/AIDS response [4].

With insecurity characterizing the crisis, fHCWs sharply perceived persistent challenges with access to and availability of health care services (HIV care, commodity supply chain) in the communities as even health care providers became targets and had to flee for safety. These effects equally had inter-relating implications on the continuity of treatment of PLWHA. Also, with the crisis came socio-economic hardship. As people adopted new life styles, adapting to new settlements, engaging in adverse health seeking and sexual behaviors, an increasing risk of new HIV infections was commonly observed. To respond to this, health promotion was strengthened, community based activities and facility-led community based approaches were employed.

Our results are particularly pertinent because while the country is clamoring for epidemic control of HIV, experiences as documented can clearly reverse years of hard work in the HIV response [17]. Additional relevance of our analysis of fHCWs’ perceptions of the effects of instability on the HIV/AIDS response is the fact that the field experiences are undiluted, straight from the horses’ mouth [4] and more so, the country’s political instability is still ongoing. In addition, though the sources represent a varied cream of health institutions including both faith-based and government-owned, they all appear to be aware and willing to discuss the implications of political instability on Cameroon’s response to HIV/AIDS and, more broadly, on the overall health system. The intensity and lack of restraint with which the respondents spoke at length about the subject, however, was palpable and beckoned for redemption.

Our findings are in line with those of other studies conducted in nations or environments with comparable levels of chronic political instability. There is a great deal of study in medical anthropology and allied fields about the connection between political unrest and health, particularly HIV/AIDS, and a large portion of this work has been carried out in sub-Saharan Africa and other places that experience political unrest [5, 6, 9, 20, 24–29]. To our knowledge, this is the first attempt to look qualitatively at the forms and impacts of political instability on the HIV/AIDS response in Cameroon. Prior HIV/AIDS research in Cameroon has been mostly biomedical in approach and outcome. More and better evidence-based HIV/AIDS research and programming may result from an understanding of how fHCWs see the phenomena of persistent political instability and the particular difficulties it offers for national HIV/AIDS responses [4, 6, 29].

Interviewees repeatedly named challenges with access to and availability of health care services (HIV care, commodity supply chain) as one of the key issues slowing down the HIV response during the crisis, pointing out erratic lockdowns, displacement of community-based HCP for fear of their lives, insecurity as a driver to constraints in access, shut down or destruction of health facilities offering decentralized minimum package of HIV care services and disruption in ARV supply and dispensation practices. In some areas, access was limited due to increased insecurity or restrictions due to the lack of identity cards. These testaments are consistent with documentation in UNAIDS’ 2015 information note, narrating that more than one million people were estimated to have been unable to access anti-retroviral therapy, due to humanitarian emergencies in 2013. It further expounded that ‘many were displaced, lacked access to essential HIV services and suffered as a result of shortages that could have been avoided’ [30]. Also, other studies have alluded to the fact that conflicts can limit drug availability due to supply chain interruptions [23, 24] and that difficulties with access and follow-up qualify the issue of ART and HIV management as a whole, more complex in humanitarian settings than in a typical resource-poor setting [31]. This underscores the road block implication that access has on the much-needed care reaching PLWHA in the context of instability.

Regarding the impact on the continuity of treatment, many respondents confessed that keeping patients on treatment in the context of the crisis was a daunting task. Citing the sharp drop in the active file of PLWHA, many respondents blamed the erratic lockdowns, spontaneous migration of clients and crisis-induced economic constraint encouraging missed appointments. Episodes of political insecurity in developing countries can quickly destabilize ART programs and lead to treatment interruptions [25]. Interruption in the continuity of treatment due to political conflicts is not infrequent in resource-limited settings. Our findings align with studies carried out in South Africa and Kenya [5, 26, 27]. Several studies, consistent with our findings [6, 27, 32] equally echoed claims of displacement of patients owing to the destruction of homes, violent environments, or lack of necessities as well as lack of available and affordable transport as a major deterrent to access to care with consequent disruption of treatment.

The dynamics of epidemics both within and between the conflicted areas of NW/SW are likely to be impacted by the migration of people and demographic shifts. The spread of new viral strains could occur if HIV-positive individuals relocate. As a result of the war, new vulnerable populations could emerge that could have limited access to medical care [29]. This was the narrative of most informants as they agreed that the increased risk of new HIV infections was a serious repercussion of the crisis. These findings stand contrary to research from sub-Saharan Africa [9], which revealed that Angola’s HIV prevalence was relatively lower than rates in other Southern African nations after the end of the conflict in 2002. These data suggest that HIV transmission may have been halted in this case by conflict. However, a study reviewing index testing practices in Mamfe - one of the most conflict-affected communities in the south west region of Cameroon, revealed a positivity rate as high as 37.2% of partners tested positive for HIV mostly within the sexually active ages of 35–44 years [22], suggestive of ongoing unchecked predisposing factors in the enclaved community [20, 27]. Also, although there is no proof that the civil war in Guinea-Bissau, which erupted in 1998, had a long-term impact on the patterns of HIV-1 or HIV-2 prevalence, it is possible that it ignited HIV-1 transmission, given that HIV-1 prevalence more than doubled between 1997 and 1999 [20]. Similarly, despite no appreciable changes in the country’s HIV/AIDS prevalence, a study in Libya revealed the spread of new HIV strains and the accumulation of HIV cases in new regions from those devastated by armed conflict [29]. Hence, to emphasize the joint burden of violent conflict and infectious disease, additional studies are therefore required.

HIV/AIDS and poverty are linked in two ways: poverty is a major factor in transmission, and HIV/AIDS can deplete people in ways that exacerbate the disease [33]. Poverty results in malnutrition, compromising the immune system thereby predisposing poorer populations to communicable diseases such as tuberculosis. In fact, malnourished people living with HIV are 2–6 times more likely to die in the first six months of treatment [30]. Conversely, PLWHA are likely to plunge into poverty, secondary to the high cost of treatment or lack of livelihood [34–36]. Unfortunately, the political instability serves to compound negatively on these syndemics, further worsening the vicious cycle for PLWHA [37]. Adapting to these hardships, increasingly, women were taking up roles as breadwinners, as father figures either displaced or were victims of the war. The key informants were able to capture these experiences, which seem to be in line with findings from Guinea Bissau [38], where respondents discussed both the dissolution of the family and the vulnerability of girls and young women as potential sources of revenue through transactional sex. Also, using the theories of collective action/responsibility and social cohesion, a different paper in the same vein, involving four nations; South Africa, Botswana, Uganda, and Zimbabwe, concludes that the dissolution of social ties brought on by various conflicts and unrest is one of the primary causes of the HIV/AIDS epidemic [6]. This highlights the complicated relationship between socio-economic depletion driven by instability, and regression of the HIV response in the country.

Insecurity in all its varieties was the main theme that emerged from the experiences of fHCWs with armed agents. Our study suggested HCWs being specifically targeted by warring parties, as almost all respondents had one or more mental imprints of vicious experiences conforming to their confessions. Our findings are in line with a systematic review of papers concerning HCWs in conflict settings, which highlighted violence against HCWs as the most discussed theme [39]. Again, similar to our observations, studies on the effects of armed conflicts on population health also supported the concept of HCWs as specifically marked elements by armed groups [37]. In fact, one Cameroonian study investigating the experiences of 12 HCWs in a remote hospital in the North West region of Cameroon with armed groups, suggested the same theme [7]. To emphasize the dire effect of this reality, an observational research in Cameroon actually estimated a total of 11 HCWs killed since the onset of the conflict [10]. Our report on the experiences of fHCWs stands in flagrant violation of the dictates of the international humanitarian law [40], which expressly prohibits targeting by armed agents on those providing medical or humanitarian assistance in a conflict setting.

Studies that are based on perceptions typically suffer from exaggeration or underestimation of problems [41]. The fact that the opinions cannot be regarded as representing all parties involved in the HIV/AIDS response may be a possible constraint. Secondly, although it did not stop interviewees from sharing vital insights with the interviewer, the interviewer’s association with the Cameroon Baptist Convention Health Services, a major HIV response implementer in both impacted regions, may have presented a limitation. All of the key informants already had relationships of trust with the interviewer. Most of the time, this trust had been developed through many years of working together to combat HIV/AIDS in Cameroon. Although this might have added bias, it probably allowed for more genuine reactions and insider perspectives. Due to the pre-existing rapport, respondents may have given information that was biased toward social desirability; nonetheless, it should be highlighted that the conclusions are based on emergent themes with to eradicate individual biases. Finally, the study’s exclusion of patients and service recipients as key informants is another factor that may not be ignored.

Further study could serve as a foundation for recommendations on how to improve national HIV/AIDS responses in low-resource, non-conflict contexts that are stymied by institutional inertia brought on by political instability. It would also be helpful for future research if there were more systematic investigations into the phenomenon of political instability’s effects on HIV/AIDS outcomes. Future epidemiological studies in Cameroon should use quantitative methods to evaluate empirically the effects of political instability on the HIV/AIDS epidemic, as Rasmussen et al. have recently done, to better understand the direct links between political instability and indicators that monitor HIV/AIDS programs [42]. Additional research should be conducted to better understand important issues, such as the prevalence and dynamics of HIV infection in the nation as demonstrated by Daw et al. [29] or the gender-related drivers and modes of HIV transmission in Cameroon, paying particular attention to the vulnerabilities of young girls and women.

Finally, our findings highlight the significance of urgently required reforms to strengthen the health sector in areas such as healthcare decentralization, supply chain, and safeguarding fHCWs from political quagmires.

## Conclusion

The performance and organization of the health system can be significantly impacted by political instability. For over half a decade, political instability has been manifested in the two English-speaking regions of Cameroon through a cycle of peaceful protests in 2016, which quickly evolved into a full-fledged armed conflict. These events have had pernicious effects on the country’s fight against HIV/AIDS and could be one of the driving factors of a higher HIV/AIDS burden. Though the national HIV prevalence seems to be on the decline since 2014, set at 2.7% in 2018 - just around the onset of the crisis, the current prevalence is likely to be higher, especially when disaggregated for the south west and north west regions. Frontline healthcare workers have identified the effects of instability as a barrier to developing a successful local response to HIV/AIDS in the two impacted regions of Cameroon. According to the results of our study, respondents were eager to express their opinions about the impact that the nation’s ongoing instability has had and is still having on the HIV/AIDS response. Any attempt to study and try to comprehend the country’s disease profile must acknowledge the significant impact that political instability has had on the response to the HIV/AIDS epidemic. Coordinated efforts should be made to strengthen the health sector in areas like HIV healthcare decentralization, supply chain, and protection of frontline healthcare workers from political snares in order to lessen the impact of the nation’s political instability on the HIV/AIDS response and, more generally, on the entire health sector.

## Data Availability

The datasets used and/or analyzed during the current study are available from the corresponding author on reasonable request.
